# Targeted Metabolomic Profiling of Peritoneal Dialysis Effluents Shows Anti-oxidative Capacity of Alanyl-Glutamine

**DOI:** 10.3389/fphys.2018.01961

**Published:** 2019-01-21

**Authors:** Florian M. Wiesenhofer, Rebecca Herzog, Michael Boehm, Anja Wagner, Markus Unterwurzacher, David C. Kasper, Seth L. Alper, Andreas Vychytil, Christoph Aufricht, Klaus Kratochwill

**Affiliations:** ^1^Christian Doppler Laboratory for Molecular Stress Research in Peritoneal Dialysis, Department of Pediatrics and Adolescent Medicine, Medical University of Vienna, Vienna, Austria; ^2^Division of Pediatric Nephrology and Gastroenterology, Department of Pediatrics and Adolescent Medicine, Medical University of Vienna, Vienna, Austria; ^3^ARCHIMED Life Science, Vienna, Austria; ^4^Division of Nephrology and Vascular Biology Research Center, Beth Israel Deaconess Medical Center, Boston, MA, United States; ^5^Department of Medicine, Harvard Medical School, Boston, MA, United States; ^6^Division of Nephrology and Dialysis, Department of Medicine III, Medical University of Vienna, Vienna, Austria

**Keywords:** N(2)-L-alanyl-L-glutamine, oxidative stress, chronic kidney disease, metabolome, methionine sulfoxide

## Abstract

Readily available peritoneal dialysis (PD) effluents from PD patients in the course of renal replacement therapy are a potentially rich source for molecular markers for predicting clinical outcome, monitoring the therapy, and therapeutic interventions. The complex clinical phenotype of PD patients might be reflected in the PD effluent metabolome. Metabolomic analysis of PD effluent might allow quantitative detection and assessment of candidate PD biomarkers for prognostication and therapeutic monitoring. We therefore subjected peritoneal equilibration test effluents from 20 stable PD patients, obtained in a randomized controlled trial (RCT) to evaluate cytoprotective effects of standard PD solution (3.86% glucose) supplemented with 8 mM alanyl-glutamine (AlaGln) to targeted metabolomics analysis. One hundred eighty eight pre-defined metabolites, including free amino acids, acylcarnitines, and glycerophospholipids, as well as custom metabolic indicators calculated from these metabolites were surveyed in a high-throughput assay requiring only 10 μl of PD effluent. Metabolite profiles of effluents from the cross-over trial were analyzed with respect to AlaGln status and clinical parameters such as duration of PD therapy and history of previous episodes of peritonitis. This targeted approach detected and quantified 184 small molecules in PD effluent, a larger number of detected metabolites than in all previous metabolomic studies in PD effluent combined. Metabolites were clustered within substance classes regarding concentrations after a 4-h dwell. PD effluent metabolic profiles were differentiated according to PD patient sub-populations, revealing novel changes in small molecule abundance during PD therapy. AlaGln supplementation of PD fluid altered levels of specific metabolites, including increases in alanine and glutamine but not glutamate, and reduced levels of small molecule indicators of oxidative stress, such as methionine sulfoxide. Our study represents the first application of targeted metabolomics to PD effluents. The observed metabolomic changes in PD effluent associated with AlaGln-supplementation during therapy suggested an anti-oxidant effect, and were consistent with the restoration of important stress and immune processes previously noted in the RCT. High-throughput detection of PD effluent metabolomic signatures and their alterations by therapeutic interventions offers new opportunities for metabolome-clinical correlation in PD and for prescription of personalized PD therapy.

## Introduction

Peritoneal dialysis (PD), as a home-based renal replacement therapy for patients with end-stage renal disease, offers an attractive (and in some aspects, superior) alternative to conventional center-based hemodialysis (Mehrotra et al., 2016; Pippias et al., 2016; van de Luijtgaarden et al., 2016; Li et al., 2017). The PD effluent contains endogenous and exogenous metabolites, proteins, nucleic acids, and free-floating cells, all available for sampling as a non-invasive liquid biopsy without burden to the PD patient. Metabolites are relatively stable and easily accessible end products of gene expression and protein activity and as the ultimate effector level of biological systems, they are predictive of cellular and tissue phenotype (Dettmer et al., 2007; Kuehnbaum and Britz-McKibbin, 2013; Aretz and Meierhofer, 2016). As such, metabolites are particularly attractive diagnostic components of PD effluent that may reflect or predict the complex clinical phenotypes and outcomes of PD patients (Herzog et al., 2018). However, the metabolome of PD effluents remains little explored.

In clinical practice, urea nitrogen, creatinine, and other selected components are measured in PD effluents and 24-h sampling of dialysate and urine, to monitor dialysis therapy efficacy, usually as part of a peritoneal equilibration test (PET) of standardized dwell time, to compare transperitoneal transport characteristics of surrogate molecules of different molecular weights. These parameters, however, reflect only a limited aspect of the complex metabolic changes produced in these patients by uremia and therapeutic intervention (Vaidya and Bonventre, 2006). Measurement of a wide range of metabolites should allow a more accurate assessment of the complex clinical phenotype of patients than allowed by the few conventional analytes.

Metabolomics techniques systematically measure multiple metabolites directly from complex biological samples (Mamas et al., 2011). Technological progress in high-resolution accurate mass (HRAM) spectrometry and full-scan capabilities theoretically permits simultaneous identification and quantitation of a great number of molecules. Capture of all sample data potentially allows unbiased, untargeted collection of information on as many *a priori* unknown metabolites as possible (Kell et al., 2005; Vanholder et al., 2015). The major drawbacks of the untargeted approach are the non-validated and at best semi-quantitative results. Despite the theoretical capability to analyze all metabolites, the important pre-analytical and technical differences of the analytes have to date hindered expansion of the PD effluent metabolome beyond ∼70 identified metabolites (Dunn et al., 2012; Guleria et al., 2014; Csaicsich et al., 2015; Vanholder et al., 2015; Aretz and Meierhofer, 2016; Kratochwill et al., 2016). The alternative targeted approach relies on a pre-defined list of candidate metabolites for which the method has been previously optimized and validated. The aim is to generate metabolic profiles under various conditions from defined cohorts of (PD) patients, generating metabolic fingerprints reflecting pathological progression stages and their modulation by therapeutic interventions.

PD fluid supplementation with glutamine, added as the stable, glutamine-releasing dipeptide, alanyl-glutamine (AlaGln), has demonstrated *in vitro* and *in vivo* restoration of cellular stress responses, immune modulation and reduced peritoneal fibrosis (Bender et al., 2010; Kratochwill et al., 2012; Herzog et al., 2014; Ferrantelli et al., 2016). Randomized controlled trials (RCTs) showed that improved cellular immune competence and restored stress responses in peritoneal cells by AlaGln supplementation of PD fluid can be translated into the clinical setting (Kratochwill et al., 2016; Herzog et al., 2017, 2018; Vychytil et al., 2018). These clinical trials allowed collection of PD effluent samples for characterization of PD-related mechanisms and their alterations by AlaGln supplementation.

In this study, we applied, for the first time, a targeted metabolomics approach to the study of PD effluent from stable patients on chronic PD therapy. The aim was to expand and validate the metabolome of PD effluent and to investigate the effects of AlaGln addition to PD fluid as a new therapeutic intervention, using a highly standardized, high-throughput method.

## Materials and Methods

### Peritoneal Dialysis (PD) Effluent Samples

Peritoneal dialysis effluent samples were obtained during a prospective randomized, open-label, two-period, cross-over phase I/II study conducted at the Department of Nephrology of the Medical University of Vienna, Austria. The study protocol was approved by the local ethics committee of the Medical University of Vienna (EK 867/2010, EK 1167/2013, EK 2035/2015), registered at www.clinicaltrials.gov (NCT01353638), and performed in accord with the Declaration of Helsinki. The study design, clinical methods, eligibility criteria, randomization, patient characteristics, and adverse events have been previously described (Kratochwill et al., 2016). In brief, 20 stable PD patients (13 male/7 female mean age 58 years) with a mean PD vintage of 2.4 years were treated per protocol. Patients were judged as clinically stable and had no severe concomitant disease (5 patients had a history of peritonitis more than 3 months prior to sample collection, 14 patients had residual renal urine output, and 6 patients were anuric). All patients provided written informed consent prior to trial participation.

Effluent samples were collected in standard 9 ml native collection tubes (Vacuette, Bio-Greiner-One, Kremsmünster, Austria) immediately after completion of instillation of the dialysis fluid into the patients’ cavity (=time point 0) and 4 h later (=time point 4 h) after each of two standard PETs (La Milia et al., 2013), one using commercially available PD fluid (Dianeal, 3.86% glucose, Baxter, Deerfield, IL, United States) and a second with the same PD fluid supplemented with 8 mM AlaGln (“Dipeptiven” = N(2)-alanyl-L-glutamine 200 mg/mL, Fresenius Kabi, Bad Homburg, Germany), performed in randomized order. The two PETs were separated by a wash-out period (28–35 days) (Supplementary Figure S1).

### PD Effluent Analyses

Peritoneal dialysis effluent was centrifuged (250 × *g*, 10 min) immediately following collection, and cell-free supernatant samples were aliquoted and stored at −80°C until further analysis.

Peritoneal dialysis effluent concentrations of glucose and creatinine were measured by validated standard methods in the clinical laboratory of the Vienna General Hospital. Creatinine concentrations were determined by a kinetic measurement of Jaffé reaction and corrected for high glucose levels by determination of a correction factor from measurements of unused PD fluid with the same method. PD effluent concentrations of glutamine and alanine were measured as previously described (Kratochwill et al., 2016) with the EZ:Faast amino acid kit (Phenomenex, Torrance, CA, United States). Amino acids were extracted by solid phase extraction, derivatized to chloroformates, dried, and reconstituted after addition of homoarginine, methionine-d3, and homophenylalanine as internal standards. Liquid chromatographic (LC) separation on an EZ:Faast AAA-MS column (250 mm × 2.0 mm) was followed by data acquisition by a triple quadrupole mass spectrometer (MS) (Waters Corp., Milford, MA, United States) and data processing by QuanLynx software v.4.1 (Waters).

### Targeted Metabolomics in PD Effluent Samples

Metabolites (*n* = 188) in PD effluent samples were analyzed using the AbsoluteIDQ p180 kit (Biocrates Life Science AG, Innsbruck, Austria). Sample preparation, analysis, data processing and validation were performed according to the manufacturers’ instructions. Internal standards, calibration standards and quality controls were dissolved and used immediately before use. Ten microliters individual PD effluents, calibration standards or quality controls were mixed with 10 μl internal standard. Phosphate buffered saline (Sigma-Aldrich, St. Louis, MO, United States) was used as blank. Samples dried 30 min at room temp. by nitrogen evaporator were derivatized with phenylisothiocyanate (in 50 μl of 1:1:1 ethanol:H_2_O:pyridine for 20 min at room temp), then dried again for 60 min. Dried samples were extracted in 300 μl methanol containing 5 mM ammonium acetate for 30 min with shaking at 450 rpm. All mixtures were filter-centrifuged, and flow-throughs were captured for analysis. Amino acids and biogenic amines were separated on a C18 column and gradient-eluted [0.2% formic acid in water (v/v) to 95% 0.2% formic acid in acetonitrile (v/v)]. For flow injection analysis (FIA) of acylcarnitines, glycerophospholipids and hexoses, the mobile phase was prepared by mixing FIA reagent (manufacturer-provided) with methanol. Samples were analyzed by MS (6500 QTRAP, AB Sciex, Framingham, MA, United States) run in multiple reaction monitoring (MRM) mode for both LC and FIA analysis. Data processing and validation used MetIDQ software (Biocrates Life Sciences AG).

### Statistical Analysis and Data Visualization

All statistical analyses and visualizations used R (v3.5.1^1^). Mean and median concentrations and corresponding standard deviations and interquartile ranges were calculated for all targeted metabolites from all samples. Correlation matrices for all metabolites were generated through hierarchical clustering by Euclidian distance. Correlation of PD effluent and plasma metabolite concentrations in 911 healthy subjects was from data of Goek et al. (2013). All features of the data set were subjected to Welch *t*-tests for changes in the following patient characteristics: (i) “Peritonitis History”: preceded episodes of peritonitis, minimum 3 month before enrollment into the study; (ii) “Anuric”: comparison of patients with residual renal function (RRF) and anuric patients; (iii) “Time on PD”: continuous PD therapy for more or less than 12 months prior to study enrollment. The effect of the study intervention (8 mM AlaGln in PD fluid during a 4 h PET) was tested using paired *t*-tests. Due to the exploratory nature of this study, no power calculation was performed; the power calculation in the trial from which the samples were obtained was aimed for a power of 80% to detect a difference in means of 30% points, using a 0.05 two-sided significance level at an assumed standard deviation of within-subject period differences of 50% points for the primary outcome parameter (Kratochwill et al., 2016). All results were Benjamini-Hochberg-corrected for multiple testing. Custom metabolic indicators in the dataset were automatically calculated from metabolite concentrations by the MetIDQ software and were included in this analysis.

### Data Availability

All datasets for this study are included in the manuscript and the Supplementary Files.

## Results

**FIGURE 1:**
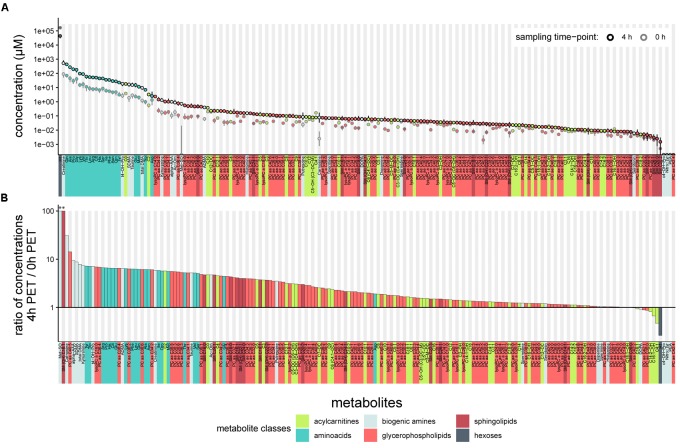
Concentrations of 188 targeted metabolites in PD effluents after a standardized 4-h peritoneal equilibration test (PET). **(A)** Median metabolite concentrations (circles) in PD effluents (standard PD fluid with 3.86% glucose). Bars above and below circles indicate upper and lower quantiles; semicircles represent undetected metabolites. Of 188 metabolites, 184 were detected after a 4 h PET dwell (black-outlined circles) and 182 in samples at the 0 h PET time point (immediately taken after initial filling of the PD fluid; gray-outlined circles). **(B)** Ratios of PD effluent metabolite concentrations after 4 h as compared to the 0 h sampling time. Ratios > 1 indicate higher levels after 4-h dwell, ratios < 1 indicate decreased concentrations after 4-h dwell as compared to 0 h, or complete absence of the metabolite at both sampling time-points (c4-OH-Pro, Nitro-Tyr, PEA, PC ae C42:4). Methionine Sulfoxide (Met-SO) and sphingomyelin SM (OH) C16:1 (see astericks “^∗∗^” above leftmost columns) were undetectable at initial sampling (0 h) and were assigned ratio values of 100. aa, in glycerophospholipids (GLP) both moieties bound by ester bonds to the glycerol backbone, ae, in GLP moieties bound by one ester and one ether bond to the glycerol backbone.

We first tested the feasibility of a targeted metabolomics approach in cell-free PD effluents. We used 10 μl effluent samples of standardized 4-h dwells from 20 patients included in a randomized controlled cross-over trial. PET samples were taken immediately after filling (0 h) and 4 h after filling (4 h). One hundred eighty four of the 188 total metabolites in the targeted panel were detectable in PD effluent samples (Figure 1 and Supplementary Table S1), and metabolites of all six metabolite classes in the panel could be quantified. The most abundant metabolite in effluents at both sampling time points was “sum of hexoses (H1)” (reflecting the 3.86% glucose PD fluid used in the clinical trial for the PET). Creatinine was the second most abundant metabolite, followed by most of the free amino acids included in the targeted assay. All measured free amino acids were found in the upper quartile of all quantified metabolites, representing the most abundant metabolite class in PD effluents (Figure 1A).

One hundred seventy five metabolites were more abundant in PD effluents after the 4 h dwell than directly after filling, whereas only nine metabolites (H1, DOPA, four acylcarnitines: C4:1, C14:2-OH, C16:1, and C16:1-OH, and three glycerophospholipids: PC ae C30:0, PC ae C42:1, and PC aa C42:2) showed higher concentrations in the PD effluent directly after filling. As expected during PD therapy, hexoses showed the strongest decrease in concentration (-3.8-fold) after 4 h dwell time (Figure 1B). Methionine sulfoxide (Met-SO) and the sphingomyelin SM (OH) C16:1 showed the highest relative increase after 4 h dwell (since they were initially undetectable) and their ratios were manually set to 100 for graphic display (Figure 1B). Next highest ratios were those of carnosine (31.2), alpha-aminoadipic acid (alpha-AAA) (9.4), hydroxyproline (t4-OH-Pro) (7.1), symmetric dimethylarginine (SDMA) (8.8), and total-DMA (7.9). Figure 1B illustrates the descending order of D4/D0 metabolite concentration ratios.

**FIGURE 2:**
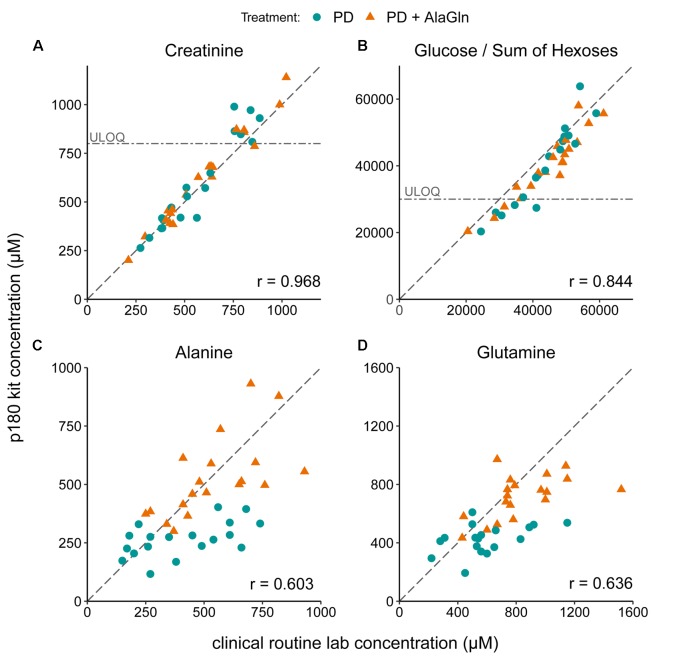
Validation of metabolomic quantitation of four metabolites by correlation with routine clinical laboratory methods. Correlations of **(A)** creatinine, **(B)** glucose, **(C)** alanine, and **(D)** glutamine concentrations with the concentrations measured in the p180 metabolomics assay (“sum of hexoses”: 90–95% glucose). Pearson’s correlation coefficients (r) are indicated. Creatinine and glucose concentrations were partly above the upper limits of quantification (ULOQ). Dashed line: line of identity for each comparison.

The PD effluent metabolite concentrations measured by the p180 assay were validated by comparison to concentrations of selected metabolites measured in the same samples by routine clinical laboratory methods (Figure 2). Targeted metabolomics values of creatinine and glucose correlated very well with measurements by validated routine clinical laboratory methods, with Pearson’s coefficients of 0.968 and 0.844, respectively (Figures 2A,B). Although creatinine and the sum of all hexoses (including glucose) concentrations were found above the kits’ respective upper limits of quantification, linearity of the assay was preserved. Alanine and glutamine were originally assessed by a LC-MS-based method, comparable to the method used in the p180 assay but with only a one-point calibration. Correlation of both measurements was still obvious with correlation coefficients for alanine and glutamine of 0.603 and 0.636 (Figures 2C,D).

**FIGURE 3:**
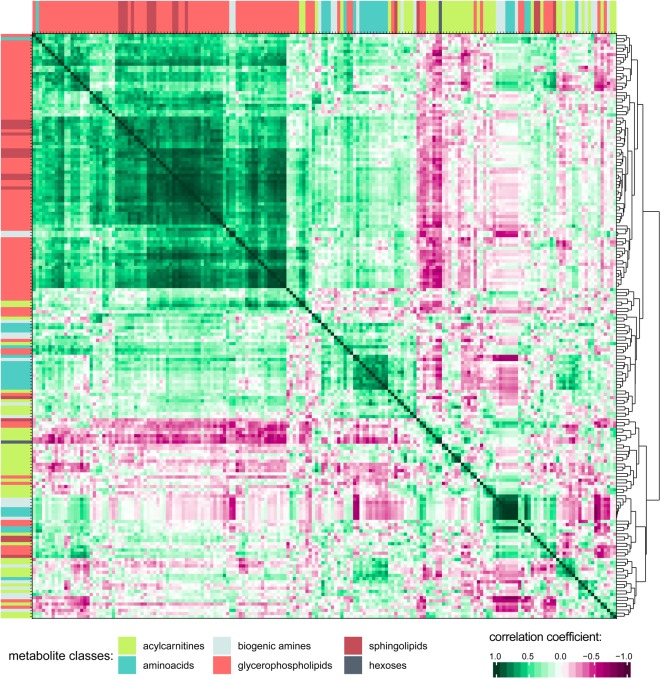
Correlation matrix of all metabolites in PD effluent after a 4 h peritoneal equilibration test (PET) dwell. Pearson’s correlation coefficients were calculated for all metabolite combinations. Only detectable (non-zero) metabolites are included in the correlation matrix. Hierarchical clustering was performed on the Euclidian distance between the metabolites’ correlation coefficients. Metabolites are colored by substance class (Supplementary Figure S2 shows this figure at higher resolution, and includes metabolite names).

Associations and similarities between individual metabolites were sought through generation of a correlation matrix (Figure 3; see Supplementary Figure S2 for a higher resolution image including metabolite names and correlation matrix of the 0 h PET data). Hierarchical clustering of the Euclidian distance between Pearson’s correlation coefficients calculated for all metabolite pairs revealed strong positive correlation for the class of glycerophospholipids (GPLs), which also correlated with most of the sphingolipids (SLs). To some extent, acylcarnitines and amino acids (AAs) are clustered together as well, although their individual patterns show more variability probably reflecting their higher chemical diversity when compared to the GPLs. Indeed, AAs form distinct subgroups, likely reflecting chemical features, as indicated by the extreme positions of the unique AAs proline and glycine. The small amino acid glycine clustered with GPLs, but also positively correlated with most other AAs. Of the included metabolic classes, the biogenic amines showed the most diverse clustering behavior.

**FIGURE 4:**
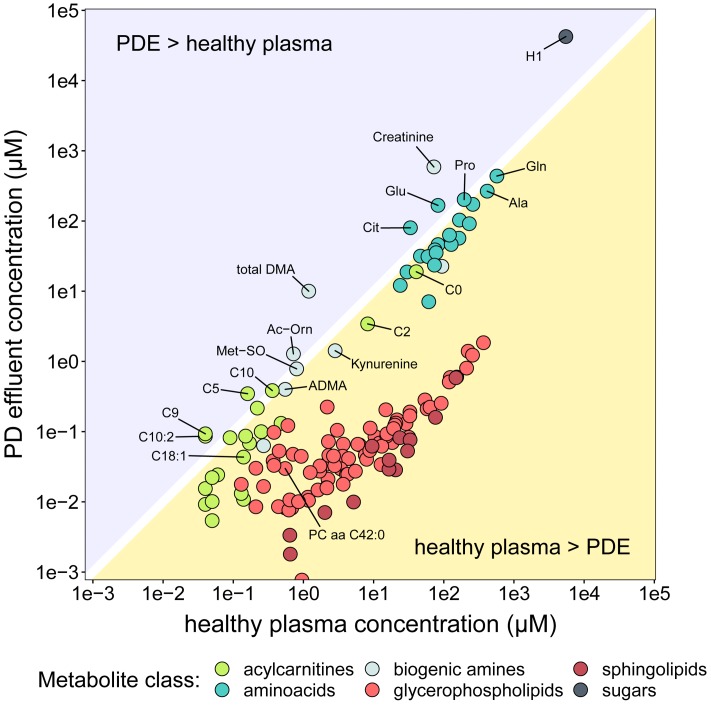
Comparison of metabolite concentrations in PD effluent and healthy plasma. The *y*-axis (log scale) shows mean concentrations of PD effluent metabolites. The *x*-axis (log-scale) shows mean concentrations of plasma metabolites in 911 healthy individuals Goek et al. (2013). Only metabolites reported in the plasma dataset are shown (*n* = 140), colored by substance class.

The PD effluent concentrations of individual metabolites reflect the sums of their plasma concentrations, their transport into the peritoneum and/or local production. To gain an impression of the systemic background of the observed metabolites, we compared PD effluent concentrations in our RCT subjects with plasma concentrations measured in a large cohort of healthy individuals using the same method (Goek et al., 2013; Figure 4). These literature derived data served as an approximation of plasma levels of respective metabolites. This crude strategy became necessary as no serum samples of studied patients were available from the RCT. As expected, PD effluent concentrations of glucose (H1; 42.3 ± 13.8 mM) and creatinine (588.6 ± 240.1 μM) after 4-h dwells exceeded those in healthy plasma. In contrast, PD effluent concentrations of most AAs and biogenic amines after a 4-h dwell were slightly below or similar to healthy plasma levels. Post-4-h PD effluent concentrations of glutamic acid (Glu), citrulline (Cit), acetylornithine (Ac-Orn), and total DMA, as well as of three acylcarnitines (C5, C9, C10:2) exceeded healthy plasma concentrations, a perhaps surprising result given the high instilled volume of PD fluid plus the additional water osmotically absorbed from the patient circulation. PD effluent concentrations of GPLs and SLs were lower than healthy plasma levels.

**FIGURE 5:**
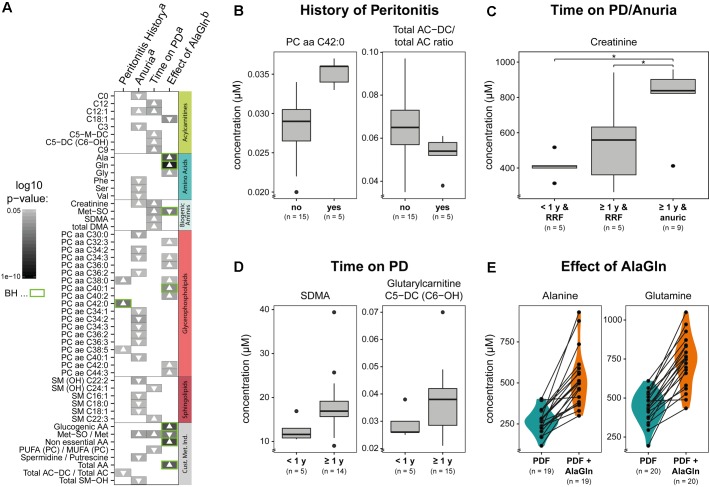
Effects on PD effluent small molecule profiles of patient factors, therapy characteristics, and PD fluid supplementation with alanyl-glutamine (AlaGln). Data shown are from PD effluents obtained after a standardized 4 h peritoneal equilibration test (PET) dwell. **(A)** Effects of **(B)** peritonitis history, **(C)** anuria, **(D)** chronic PD duration (< or ≥1 year), and **(E)** effect of 8 mM AlaGln supplementation of PD fluid (vs. standard PD fluid in one 4 h PET). All metabolites with significant results in at least one comparison are shown panel **(A)** with selected comparisons presented in detail panels **(B–E)**. Results from all *t*-tests are reported as gray-scale tiles with darker colors representing higher significance (highest *p*-value 0.049), with Benjamini-Hochberg-corrected *p*-values (green: BH < 0.05) **(C)** Kruskal-Wallis: χ^2^ = 6.93, *p* = 0.049; Dunn’s *post hoc* test between groups: <1 year and RRF vs. ≥1 year and RRF *p* = 0.1506; ≥1 year and RRF vs. ≥1 year and anuric *p* = 0.0440 (^∗^); <1 year and RRF vs. ≥1 year and anuric *p* = 0.0078 (^∗^); a, Welch *t*-tests; b, paired *t*-tests, to investigate the effect of AlaGln supplementation in the cross-over study. Met-SO, methionine sulfoxide; SDMA, symmetric dimethylarginine; total DMA, symmetric and asymmetric dimethylarginine; PUFA (PC)/MUFA (PC), ratio of polyunsaturated to mono-unsaturated glycerophospholipids; AC-DC, dicarboxy acylcarnitines; AA, amino acids; RRF, residual renal function.

**FIGURE 6:**
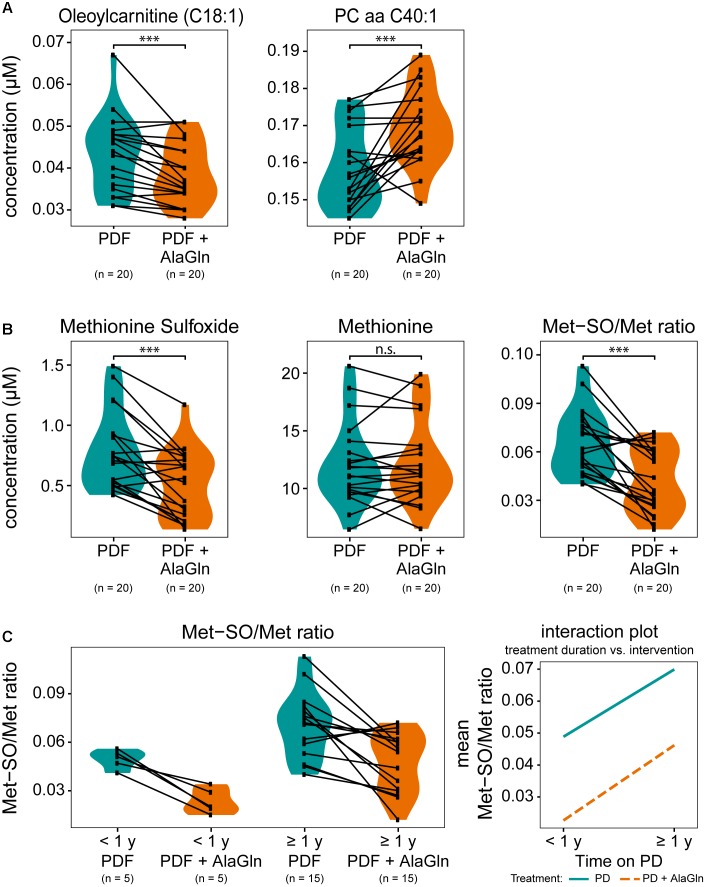
Effects on selected small molecule concentrations of alanyl-glutamine (AlaGln) addition to PD fluid (PDF). Effects of standard PDF vs. 8 mM AlaGln supplemented PDF in 4 h PET effluents compared by paired *t*-tests (except **C**). **(A,B)** All *p*-values corrected for multiple testing by BH method; ^∗∗∗^*p* < 0.001; n.s.: *p* = 0.87 **(C)** mixed design ANOVA: chronic PD duration (shorter or longer than 1 year) compared among patients and treatment intervention with AlaGln tested within individual patients; effect of PD treatment duration: *p* = 0.007; effect of AlaGln supplementation: *p* < 0.001; interaction: *p* = 0.825; Met-SO, methionine sulfoxide.

We next examined the possible influences of patient factors or AlaGln supplementation of PD fluid during the PET in the RCT on individual PD effluent metabolites and custom metabolic indicators. Patient-related variables included (i) preceding episodes of peritonitis, (ii) residual renal function (RRF) and anuria, and (iii) elapsed time since PD treatment initiation (two groups continuously treated for < or ≥12 months duration). Fifty one metabolites were significantly influenced by at least one tested variable (Figure 5 and Supplementary Table S2). A history of peritonitis was associated with changes in four metabolites: elevation of three GPLs, most pronounced for PC aa C42:0 (significant after correction for multiple testing), and reduction of the ratio of non-dicarboxy-acylcarnitines to total acylcarnitines (total AC-DC/total AC), an indicator of ω-oxidation of fatty acids (Figure 5B). Anuria was associated with significant changes in all metabolite classes. PD effluent levels of two short-chain acylcarnitines, 14 GPLs and SLs and three AAs (phenylalanine, serine, valine) showed lower concentrations than those in patients with RRF. Elevated PD effluent creatinine concentrations were associated with both time on PD treatment and with anuria. Upon closer inspection, the increase in creatinine by prolonged treatment could be explained by those patients without RRF (Figure 5C). None of the PD patients treated for less than 1 year was anuric, whereas anuria characterized 40% of PD patients treated for more than 1 year (Kruskal–Wallis: χ^2^ = 6.93, *p* = 0.049; Dunn’s *post hoc* test between groups: <1 year and RRF vs. ≥1 year and RRF *p* = 0.1506; ≥1 year and RRF vs. ≥1 year and anuric *p* = 0.0440; <1 year and RRF vs. ≥1 year and anuric *p* = 0.0078). PD effluent from patients treated with PD for more than 1 year contained higher concentrations of the biogenic amines Met-SO, SDMA and the total amount of DMA, in addition to creatinine. Levels of five acylcarnitines and one SL (sphingomyelin C22:3) were also increased with longer time on PD therapy (Figure 5D). AlaGln supplementation of the PD fluid used in the PET increased post-4-h dwell concentrations of alanine and glutamine in PD effluent (and calculated metabolic indicators that include alanine and glutamine) compared to those from the same patients after control PET with standard PD fluid (Figure 5E), but did not influence levels of other AAs, such as glutamic acid (paired *t*-test: *p* = 0.33) (Supplementary Figure S3). AlaGln-supplemented PD fluid increased post-dwell PD effluent concentrations of 8 GPLs, most prominently PC aa C40:1 (significant after correction for multiple testing), but decreased post-dwell levels of the long-chain acylcarnitine oleoylcarnitine, C18:1 (Figures 6A,B). AlaGln-supplemented PD fluid also showed decreased PD effluent Met-SO concentrations and decreased Met-SO-to-methionine ratio (Met-SO/Met), an indicator of systemic oxidative stress. The effect of AlaGln in PD fluid was opposed to the effect of anuria and time on PD, where Met-SO and Met-SO/Met levels were significantly increased. The levels of free unmodified methionine remained constant (Figure 6B). In a subgroup analysis, the AlaGln supplementation-associated decrease of the Met-SO/Met ratio was independent of PD therapy duration [mixed design ANOVA, time on PD between patients (*p* = 0.007) and AlaGln treatment within patients (*p* < 0.001); interaction (*p* = 0.825)] (Figure 6C).

## Discussion

This study reports the first targeted metabolomics analysis of PD effluent. Our approach enabled detection of up to 184 small molecules in each patient’s PD effluent obtained from standardized PETs during an RCT (Kratochwill et al., 2016) for characterization of the metabolic status during PD treatment. To our knowledge, this study presents the highest number of metabolites yet detected and quantitated in PD effluents.

Emerging technologies now allow acquisition, analysis and clinical utilization of omics level information in chronic kidney disease (CKD), end-stage renal disease (ESRD) and dialysis patients. Genomics, transcriptomics, proteomics, and metabolomics have already demonstrated value in CKD patient classification (Rossing et al., 2008; Rhee et al., 2010; Choi et al., 2011; Zhao, 2013; Mullen et al., 2014; Kottgen et al., 2018). Metabolites, as intermediates and end points of gene expression and protein activity and the sum of biotic and abiotic perturbations, represent the ultimate level of biological effectors (Kell et al., 2005; Kuehnbaum and Britz-McKibbin, 2013; Barrios et al., 2016).

Although effluents from PD patients are easily available bio-fluids, PD metabolomics remains in early stage investigation, with fewer than 80 metabolites identified to date in PD effluent (Dunn et al., 2012; Guleria et al., 2014; Tang et al., 2014; Csaicsich et al., 2015; Vanholder et al., 2015; Kratochwill et al., 2016). Considering the effort required to overcome the challenge of high abundance protein species posed by the PD effluent soluble proteome, and the low peritoneal cell number available for phenotyping, metabolites seem particularly attractive components of the PD effluent (Aufricht et al., 2017; Herzog et al., 2017, 2018). PD effluent metabolite levels reflect the sum of uremia-induced alterations of metabolism, nutrition, muscle/energy wasting and accumulation of toxic metabolites in dialysis patients, many likely functions of patient pathophysiological status, morbidity and mortality. PD effluent parameters currently monitored in clinical practice, including urea nitrogen and creatinine, reflect only limited aspects of the metabolic complexity of uremia and related therapeutic interventions (Vaidya and Bonventre, 2006; Zhao, 2013). Numerous additional small molecules may act as renal/uremic toxins, contributing directly to uremic pathophysiology, as they accumulate along with other low molecular weight substances in the setting of renal impairment (Barrios et al., 2016). Evaluation of a wider range of metabolites should therefore allow a more accurate assessment of the complex clinical phenotypes of PD patients.

The human metabolome comprises at least several thousand predicted and/or experimentally observed chemical entities varying in abundance and physicochemical properties (incl. size, molecular weight, polarity, hydrophobicity) (Aretz and Meierhofer, 2016; Wishart et al., 2018). Our previous pilot study applying untargeted metabolomics identified 41 small molecules in PD effluents (Csaicsich et al., 2015), suggesting the untargeted approach failed to deliver the systems-level view of the metabolome suggested by its name. In contrast, the targeted approach seeks not to identify unknown metabolites, but instead, screens for a pre-defined list of metabolites with pre-optimized methods and appropriate standards allowing absolute quantification (Vanholder et al., 2015; Barrios et al., 2016; Kalim and Rhee, 2017).

In this study we analyzed small molecules in cell-free PD effluent sampled at the start and the end of a standardized 4 h PET using the p180 assay, a highly reproducible, high-throughput FIA/LC-MS method already validated by multiple laboratories with plasma and serum samples (Siskos et al., 2017). Applying this technology to 10 μl volumes of individual PD effluents, we detected and quantitated 184 metabolites of the 188 included in the targeted panel. Three of the four undetected metabolites, biogenic amines *cis*-hydroxyproline, nitrotyrosine, and phenylethylamine, are at or below detection limits in healthy plasma (Siskos et al., 2017).

We validated the p180 assay results in PD effluent by comparison to previously published RCT data obtained by routine clinical laboratory methods. PD effluent creatinine and glucose concentrations measured by routine methods and by the p180 assay exhibited correlation coefficients >0.8. Effluent concentrations of alanine and glutamine previously measured by a different LC-MS-based kit (Kratochwill et al., 2016) also correlated well with the p180 assay data. In contrast to the earlier used method, which employed one-point calibration, all free amino acids are quantitated via calibration curves in the p180 assay.

The lack of plasma metabolomic measurements prevented comparison of PD effluent metabolite concentrations with systemic values from the same individuals. We therefore compared our effluent data to previously published p180 plasma metabolomics data from a population-based trial with 911 healthy participants (Goek et al., 2013). We hypothesized that abundant, peritoneal membrane-permeant plasma metabolites would partially or completely equilibrate by the end of the 4-h PET dwell, whereas plasma metabolites enriched in PD effluent would represent molecules subject to preferential peritoneal membrane transport or clearance, molecules elevated in CKD or PD, and/or locally produced molecules. However, the inferable conclusions from these results are challenged by the limitation that plasma from non-uremic individuals serves only as a rough approximation. Uremia itself causes high plasma concentrations of many metabolites, like creatinine, and patients with renal failure are likely to have metabolomic signatures, distinct from normal plasma (Breit and Weinberger, 2016). Unfortunately, no datasets (tabulated raw data) from targeted metabolomics experiments in CKD patients were available in literature. Therefore, the performed comparisons cannot be used to reliably distinguish between peritoneal transport of a solute or local production in the respective patient but only as a first insight into the PD effluent metabolome in comparison to generalized systemic levels. In future studies it will be a prime goal to obtain metabolomic data from plasma and PDE samples from the same patients in parallel. Since glucose is the major constituent of the PD fluid used in this trial, it was expected to be more abundant in PD effluent than in healthy plasma samples. PD effluent concentrations of creatinine, the best established biomarker of renal failure progression, were also higher than plasma creatinine concentrations in the healthy cohort. Post-dwell effluent concentrations of most of the smaller metabolites (AAs and biogenic amines) were only slightly below healthy plasma concentrations, in contrast to the larger GPLs and SLs. However, post-dwell PD effluent concentrations of citrulline, acetylornithine (Ac-Orn), glutamic acid, total DMA and three acylcarnitines (C5, C9, C10:2) were higher than those in healthy plasma. Acylcarnitines are elevated in cardiovascular disease, insulin resistance, as well as in CKD, in which accumulation has been routinely attributed to impaired renal clearance. Acylcarnitines are formed from carnitine and acyl-CoA and serve as fatty acid transport cofactors across mitochondrial membranes. The esterification of acyl-CoA, overaccumulated in renal failure, with carnitine to generate acylcarnitines has been proposed to retard progression of renal disease (Kerner and Hoppel, 2000; Mai et al., 2013; Barrios et al., 2016; Breit and Weinberger, 2016; Strand et al., 2017). In our cohort of PD effluent samples we found seven short and medium-chain acylcarnitines significantly elevated in association with anuria and prolonged time on PD, all of which were previously correlated with eGFR decline in a large cross-sectional study (Goek et al., 2012). In that study glutarylcarnitine [C5-DC(C6-OH)] showed the strongest effect size. This metabolite, which in our dataset correlated with duration of PD therapy, was also reported to increase in plasma in parallel with CKD progression in another CKD cohort (Nkuipou-Kenfack et al., 2014).

Also detected in PD effluent, by our approach, were the more hydrophobic, long-chain acylcarnitines which are derived exclusively from fatty acid metabolism and are regarded as markers of mitochondrial fatty acid oxidation. As these accumulated long-chain acylcarnitines are not cleared by hemodialysis (HD) (Kalim et al., 2013; Makrecka-Kuka et al., 2017), we speculate that their removal during PD may represent a previously undescribed PD-specific advantage in clearance of accumulated metabolites from ESRD patients.

Amino acids and biogenic amines were among the most abundant metabolites in PD effluent and, with few exceptions, were highly enriched in effluent during the 4-h dwell time. Clustering in the cross-correlation matrix revealed distinct sub-groups likely reflecting similar chemical properties. Kynurenine and tryptophan, the latter being degraded to hydroxykynurenine, are among the metabolites with the highest concentration increase after a 4 h PD dwell, confirming our previous untargeted analysis (Csaicsich et al., 2015). Interestingly, however, PD effluent kynurenine concentrations were not higher than those in healthy plasma, and were unassociated with any investigated clinical factors. PD effluent concentrations of phenylalanine, serine and valine were lower in anuric patients than in those with RRF. Indeed, anuria was associated with significant changes in all metabolite classes, and was the driving factor for increased PD effluent creatinine concentrations.

Arginine metabolites ADMA, SDMA, citrulline, and ornithine have been previously described as plasma metabolome markers of CKD progression and eGFR decline (Ceballos et al., 1990; Goek et al., 2013; Shah et al., 2013; Duranton et al., 2014; Nkuipou-Kenfack et al., 2014; Breit and Weinberger, 2016). Our study also found PD effluent concentrations of SDMA and total-DMA (sum of ADMA + SDMA) significantly increased with longer time on PD therapy. SDMA levels were highly correlated with creatinine levels in a meta-analysis of 18 studies encompassing 2100 patients (Bode-Boger et al., 2006; Kielstein et al., 2006). ADMA concentrations have been reported to be elevated in plasma of PD patients, but significantly lower than in HD patients, likely reflecting superior ADMA clearance by PD (Kielstein et al., 1999; Cross et al., 2001; Mittermayer et al., 2005; Zhang et al., 2010). ADMA inhibition of endothelial nitric oxide synthase (eNOS), which converts arginine to citrulline and nitric oxide (NO), may explain the higher citrulline levels in PD effluent than in healthy plasma.

Peritoneal cavity glutamine levels during PD may be sub-physiological (Kratochwill et al., 2016), a deficiency that may be associated with increased peritoneal vulnerability due to inadequate cellular stress responses and impaired metabolic and immune competence (Wischmeyer, 2002; Roth, 2008; Kratochwill et al., 2012).

In the present study we tested whether the addition of alanyl-glutamine (AlaGln) to PD fluid influences metabolite concentrations in PD effluents. AlaGln supplementation of PD fluid has already been shown to improve survival of mesothelial cells, boost peritoneal immune-competence and counteract deleterious effects of PD therapy (Kratochwill et al., 2012, 2016; Ferrantelli et al., 2016; Herzog et al., 2017, 2018; Vychytil et al., 2018). The samples used in our current study were from a previously reported randomized controlled cross-over trial testing the safety and efficacy of addition of 8 mM AlaGln to PD fluid during a single 4-h PET dwell (Kratochwill et al., 2016). Consequently, PD fluid supplementation with AlaGln dipeptide resulted in significant increases of alanine and glutamine levels in PD effluent compared to standard PD fluid. These results were confirmed by our targeted metabolomics approach. Except for a minor increase in glycine levels, AlaGln addition did not influence levels of other AAs such as glutamic acid. Glutamine is both the most abundant free amino acid of plasma and classified as conditionally essential. Besides its role in nutrition, glutamine regulates immune cell functions, glucose metabolism and glutathione-mediated redox potential (Curi et al., 2007; Roth, 2008).

AlaGln in PD fluid increased post-dwell PD effluent concentrations of 8 GPLs and one acylcarnitine. In contrast, the long-chain acylcarnitine oleoylcarnitine (C18:1) was decreased in AlaGln-supplemented post-dwell PD effluent. This acylcarnitine has not been significantly associated with CKD progression in the above-mentioned metabolomic studies but has been described as a marker of uremic cardiovascular risk in a large HD cohort (Kalim et al., 2013). The effect of its decrease in PD effluent must await further study.

Reductions of methionine sulfoxide (Met-SO) and the Met-SO-to-methionine ratio were observed in effluents from patients treated with AlaGln-supplemented PD fluid. This reduction by AlaGln was highly significant and independent of time on PD therapy, whereas longer PD vintage *per se* significantly increased Met-SO. Post-translational methionine sulfoxidation usually leads to impairment or loss of protein function. Met-SO and its ratio to unmodified methionine have been studied as markers of oxidative stress in CKD (Thornalley and Rabbani, 2014; Breit and Weinberger, 2016). The importance of oxidative stress in CKD and PD has been extensively described (Himmelfarb et al., 2002; Ksiazek et al., 2007; D’Apolito et al., 2010; Sung et al., 2013). In healthy controls renal clearance of Met-SO is low, probably reflecting intact systemic metabolism to unmodified methionine by Met-SO reductases. Oxidative stress and generation of Met-SO increase with CKD progression, paralleled by additional accumulation of Met-SO due to loss of renal clearance (Thornalley and Rabbani, 2014; Breit and Weinberger, 2016). Increased free radicals as markers of oxidative stress in PD patients were associated with decline of RRF and with technique failure (Morinaga et al., 2012).

In summary, we have demonstrated the feasibility of targeted metabolomics in minute amounts of PD effluents as a new tool to analyze a wide range of small molecules in a single measurement. The analysis of absolute abundances, of ratios between concentrations at the end and the start of the PET dwell, and comparison to plasma reference concentrations showed that the investigated panel of metabolites associated with multiple metabolic pathways can provide information of potential clinical relevance. This includes assessment of the patient’s uremic status and uremia-induced metabolic changes, of dialysis quality, of risk for PD-induced co-morbidities, and of success of therapeutic interventions. The assay shows excellent technical stability and allows comparison of metabolite concentration to external datasets, potentially increasing understanding of PD-related pathomechanisms. The behavior during a PD dwell of several known metabolites was confirmed, and other metabolites previously unknown in PD effluent were discovered to be enriched or associated with PD vintage. PD fluid supplementation with AlaGln in a randomized controlled cross-over trial reduced metabolomic markers of oxidative stress, indicating an anti-oxidative effect of the additive. The targeted metabolomics approach might represent a promising tool not only for basic and translational research but eventually also for clinical practice. Further studies will focus on defining metabolomic signatures of known and novel pathomechanisms in larger cohorts and in cohorts with extended PD duration using AlaGln-supplemented PD fluids.

## Author Contributions

FW analyzed and interpreted the data, and prepared the manuscript. RH performed the clinical trial, analyzed and interpreted the data, and prepared the manuscript. MB performed the clinical trial. AW and DK performed the MS analysis. MU analyzed the data. SA interpreted the data and critically read the manuscript. AV performed the clinical trial and critically read the manuscript. CA conceived the study, interpreted the data, and critically read the manuscript. KK analyzed and interpreted the data, prepared the manuscript, and conceived the study.

## Conflict of Interest Statement

CA is cofounder of Zytoprotec GmbH, a spin-off of the Medical University Vienna that holds the patent “Carbohydrate-based peritoneal dialysis fluid comprising glutamine residue” (International Publication Number: WO 2008/106702 A1). RH, AW, MU, and KK are former employees of Zytoprotec GmbH. AV has received honoraria and travel grants from Baxter and Fresenius (manufacturers of dialysis solutions) unrelated to this trial. DK is CEO of ARCHIMED Life Science. The remaining authors declare that the research was conducted in the absence of any commercial or financial relationships that could be construed as a potential conflict of interest. The handling Editor declared a past collaboration with the authors.

## Footnotes

1 http://www.r-project.org/
