# Supplementary material for: Targeted Metabolomic Profiling of Peritoneal Dialysis Effluents Shows Anti-oxidative Capacity of Alanyl-Glutamine
Source: Front Physiol. 2019 Jan 21;9:1961. doi: 10.3389/fphys.2018.01961 (PMC6348277; doi:10.3389/fphys.2018.01961)
Supplement: Supplementary file 1 [file Data_Sheet_1.PDF]

Supplemental Material

Targeted Metabolomic Profiling of Peritoneal Dialysis Effluents shows Anti-Oxidative Capacity of Alanyl-Glutamine

Florian M. Wiesenhofer[1,2#], Rebecca Herzog[1,2#], Michael Boehm[1], Anja Wagner[1,2], Markus Unterwurzacher[1,2], Seth L. Alper[3,4], Andreas Vychytil[5], Christoph Aufricht[2], Klaus Kratochwill[1,2*]

[1] Christian Doppler Laboratory for Molecular Stress Research in Peritoneal Dialysis, Department of Pediatrics and Adolescent Medicine, Medical University of Vienna, Vienna, Austria

[2] Division of Pediatric Nephrology and Gastroenterology, Department of Pediatrics and Adolescent Medicine, Medical University of Vienna, Vienna, Austria

[3] Division of Nephrology and Vascular Biology Research Center, Beth Israel Deaconess Medical Center, Boston, MA, USA

[4] Department of Medicine, Harvard Medical School, Boston, MA, USA

[5] Division of Nephrology and Dialysis, Department of Medicine III, Medical University of Vienna, Vienna, Austria

# these authors contributed equally to this work

Running title: The peritoneal dialysis effluent metabolome

***Corresponding author:**

Priv.Doz. DI Dr. Klaus Kratochwill

Division of Pediatric Nephrology and Gastroenterology, Department of Pediatrics and Adolescent Medicine, Medical University of Vienna

Währinger Gürtel 18-20, AT-1090 Vienna

Phone: +43/1/40400-73747

Fax: +43/1/40400-73598

E-mail: klaus.kratochwill@meduniwien.ac.at

Supplemental Figure S1: Schematic representation of the study design of the randomized controlled trial testing alanyl-glutamine (AG) in peritoneal dialysis (PD) fluids. EOS: end of study

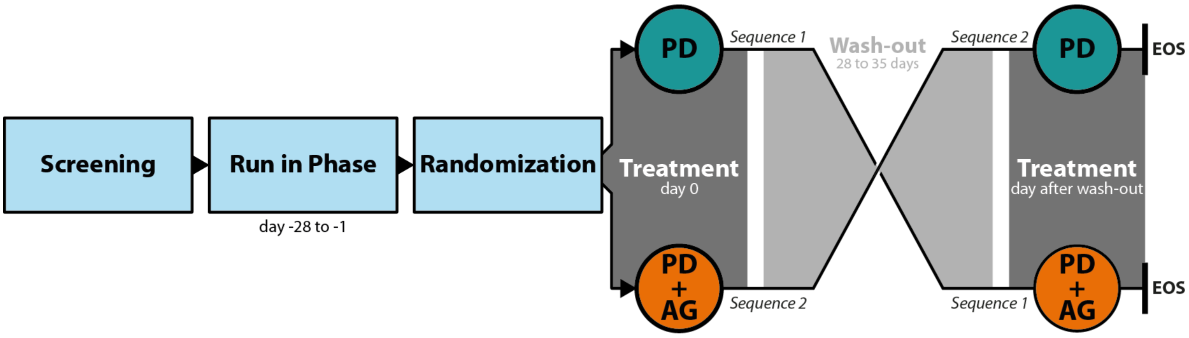

Supplementary Figure 2A+B: see high resolution files

Supplemental Figure S3: Influence of alanyl-glutamine (AlaGln) addition to PD fluid (PDF) on small molecules. Effects of 8 mM AlaGln in PDF *vs.* standard PDF in 4h PET effluents. Detailed representation of additional metabolites and custom metabolic indicators. Paired t-tests were used to compare the treatments. **A)** one outlier pair was excluded (> 200 SDs above the mean).

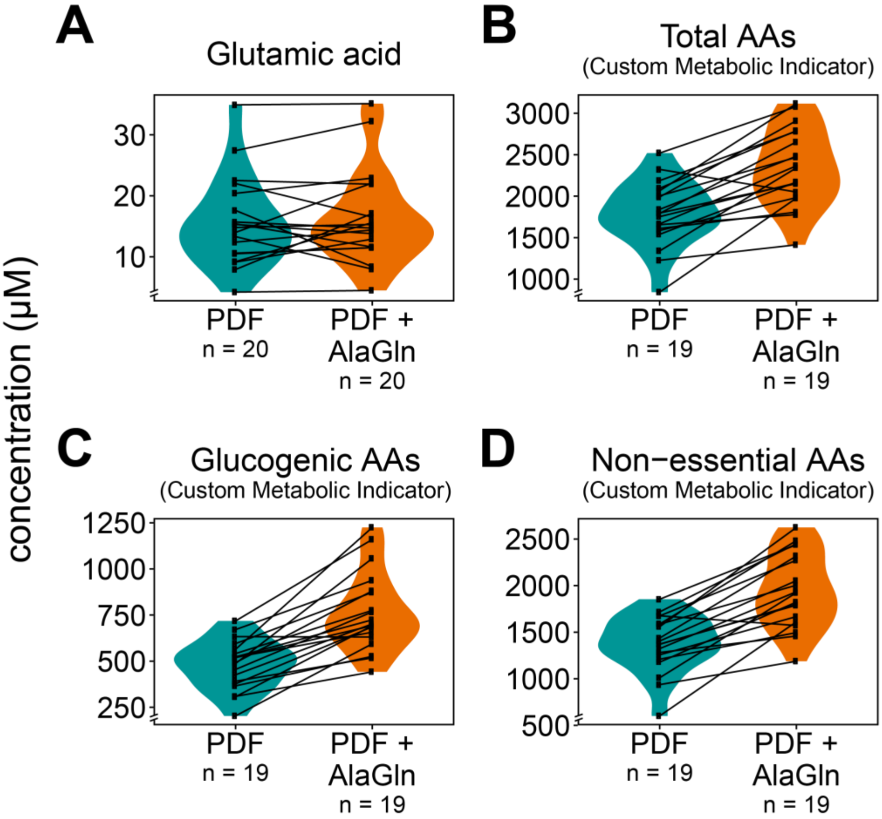

Supplemental Tables:

Supplemental Table S1: All metabolites

Supplemental Table S2: Significant metabolites with values and *p* values and BH-corrected *p* values.
