# Supplementary material for: Targeted Metabolomic Profiling of Peritoneal Dialysis Effluents Shows Anti-oxidative Capacity of Alanyl-Glutamine
Source: Front Physiol. 2019 Jan 21;9:1961. doi: 10.3389/fphys.2018.01961 (PMC6348277; doi:10.3389/fphys.2018.01961)
Supplement: Supplementary file 2 [file Data_Sheet_2.PDF]

Supplemental Figure S2 A: Correlation matrix of all metabolites in PD effluent after a 4 hour peritoneal equilibration test (PET) dwell. Pearson's correlation coefficients were calculated for all metabolite combinations. Only detectable (non-zero) metabolites are included in the correlation matrix. Hierarchical clustering was performed on the Euclidian distance between the metabolites' correlation coefficients. Metabolites were colored according to substance classes. (Supplement Figure to Figure 3).

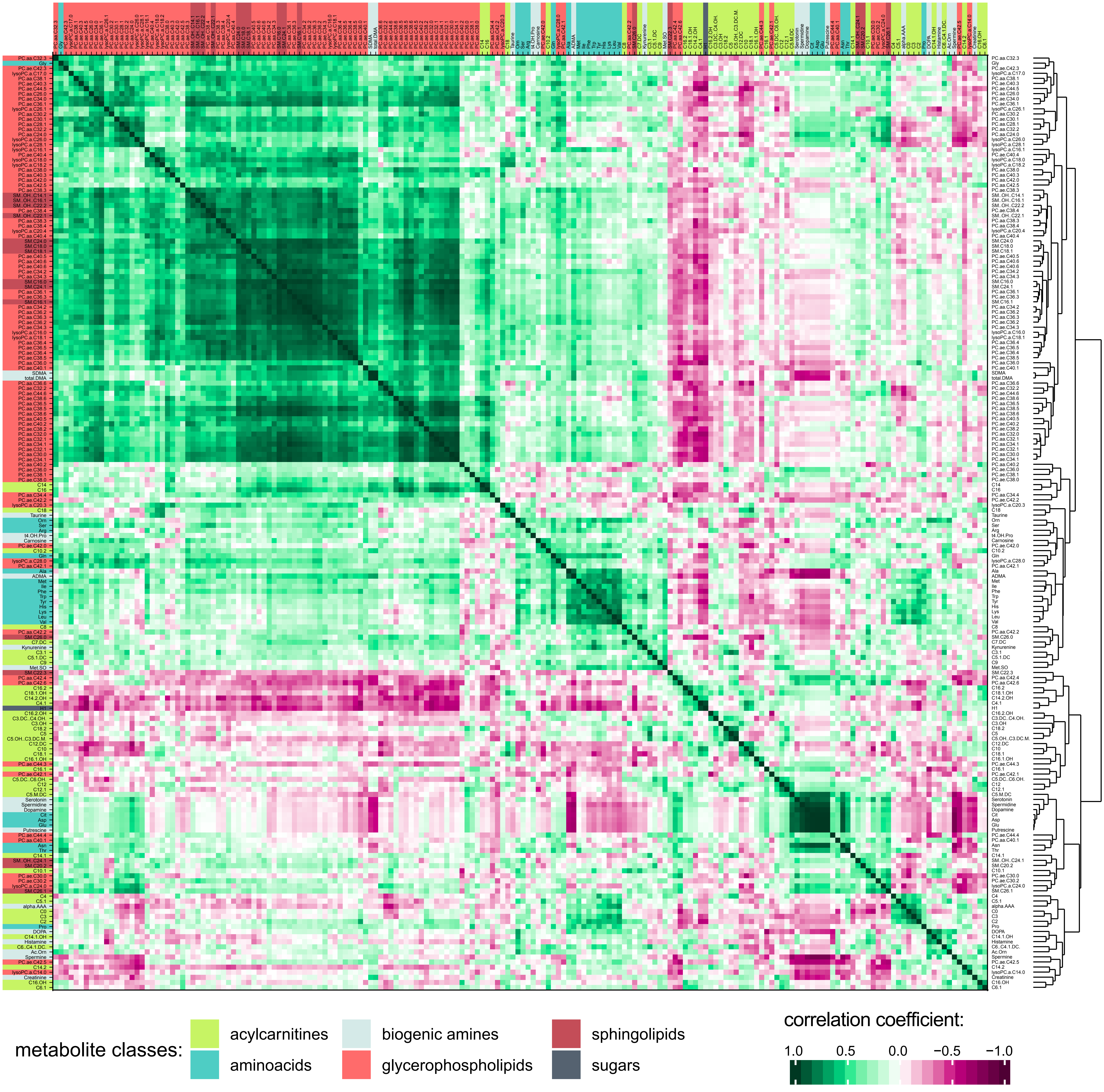

Targeted Metabolomic Profiling of Peritoneal Dialysis Effluents shows Anti-Oxidative Capacity of Alanyl-Glutamine, Wiesenhofer & Herzog, et al.
